# Nutritional Methods to Decrease N Losses from Open-Dirt Feedlots in Nebraska

**DOI:** 10.1100/tsw.2001.364

**Published:** 2001-11-21

**Authors:** Galen E. Erickson, Terry J. Klopfenstein

**Affiliations:** Department of Animal Science, University of Nebraska, Lincoln 68583-0908, USA

**Keywords:** nitrogen, volatilization, cattle feedlots, C:N ratio, carbon

## Abstract

Nitrogen (N) losses from cattle feedlots are of concern due to loss of valuable N and enrichment of the atmospheric N pool. Nutritional methods to decrease such losses would have economic and environmental benefits. One method to decrease N losses is by increasing carbon (C) on the pen surface. The most cost effective method of decreasing N losses with C may be feeding diets lower in digestibility compared to adding C directly to pens. Therefore, three experiments evaluated feeding corn bran (which is less digestible than corn) as either 0, 15, or 30% of the diet. The 15- and 30%-bran diets increase organic matter (OM) excretion by approximately 0.5 and 1.0 kg per steer per day, respectively. Compared with no bran, feeding 15 and 30% decreased feed efficiency by 7.8 and 10.4%, respectively. Nutrient balance was assessed in two trials from October through May and in one trial from June to September. During the trials from October to May, N losses were decreased by 14.5 and 20.7% for the 15- and 30%-bran diets compared with no bran. Feeding 15 or 30% bran did not influence N losses in the experiment from June to September. Increasing the C:N ratio of manure prior to cleaning open-dirt feedlots had variable results depending on time of year.

### INTRODUCTION

Nitrogen (N) emissions from livestock production are of concern for producers. When large losses occur, it is detrimental to water resources and also decreases the fertilizer value of livestock manure. N volatilization (primarily as ammonia [$NH_3$]) estimates from open-dirt feedlots range from 30 to 70% of the N that is excreted[1,2].

One method to decrease $NH_3$ emissions is to increase the carbon to nitrogen (C:N) ratio of manure. Dewes[3] added straw to cattle manure and decreased N losses from 23.2 to 5.1% of the initial N over 14 days. Others have decreased N losses from pig slurry by adding C[4,5]. Immobilization of N during composting has been enhanced by adding C to feedlot manure[6]. Adding C to manure decreases N losses by lowering pH when stored anaerobically[7] or by microbial immobilization when stored aerobically[6].

One method to increase the C:N ratio of manure is by feeding diets lower in digestible organic matter (OM), but this conflicts with the principles of diet formulation in use today. Corn bran is a fibrous byproduct of the corn wet milling industry that

[1] Published with the approval of the Director as paper no. 13431, journal ser., Nebraska Agric. Res. Div.

contains high concentrations of neutral detergent fiber[8] that is readily digested[9] but has lower digestibility than corn[10]. Corn bran may maintain animal performance when fed at 15 to 30% of diet dry matter (DM)[11]. Therefore, the objectives of these experiments were to determine if increasing dietary corn bran in beef finishing diets would increase C excretion, decrease N losses by increasing C excretion, and maintain animal performance.

### EXPERIMENTAL METHODS

#### Feedlot Performance

Three experiments were conducted consecutively to assess the impact of increasing dietary corn bran on animal performance and mass balance of N. Experiment 1 utilized 96 yearling steers (initial body weight [BW] = 385 ± 15.6 kg) fed for 128 days from October 5, 1999 until February 9, 2000. In experiment 2, 96 steers (initial BW = 408 ± 19.7 kg) were fed from February 10 until May 24, 2000 or 105 days. In experiment 3, 96 steer calves (initial BW = 420 ± 20.5 kg) were fed from June 2 until September 19, 2000 or 110 days. As indicated, all three experiments were conducted consecutively spanning 343 days with large, yearling steers.

In each experiment, steers were randomly assigned (eight steers per pen) to one of three treatments (four pens per treatment). Treatments consisted of three different diets (Table 1) in each experiment that contained either 0 (0-bran), 15 (15-bran), or 30% (30-bran) corn bran as a percentage of diet DM. Diets were evaluated using a National Research Council (NRC)[12] model to ensure adequate degradable intake protein and metabolizable protein for 420-kg steers. The goal was to utilize the NRC model so dietary supply would meet protein requirements during the feeding period while minimizing excess protein. If protein was supplied in excess of requirements, the excess supply was equivalent in grams per day across all treatments.

Animal performance was monitored due to its importance in animal production systems. Methods used for collection of performance data were typical of Nebraska production systems. Initial weight was based on two consecutive day weights recorded prior to feeding following a 5-day limit fed period. Steers were implanted on day 27 with Revalor-S® (Intervet Inc., Somerville, NJ) in experiment 1. In experiment 2, steers were implanted with Revalor-S® on day 19. In experiment 3, steers were implanted initially with Revalor-S® on day 1.

Cattle were adapted to finishing diets by replacing alfalfa hay with dry-rolled corn (DRC). Roughage was provided from both corn silage and alfalfa. Roughage levels during adaptation were 45, 35, 25, and 15% fed for 3, 4, 7, and 7 days, respectively. Steers on the 15- and 30-bran treatments were adapted similarly except that corn bran was included at target levels of either 15 or 30% during the entire 21-day adaptation period. Corn silage was the only roughage source in finishing diets and was included at 15% of diet DM. Corn silage was assumed to contain 50% grain and 50% roughage on a DM basis.

When animals were visually appraised as finished, they were marketed to a commercial abattoir (IBP Inc., West Point, NE). At slaughter, hot carcass weights were recorded and used to determine final weights assuming a common dressing percentage (62). Following a 24-h chill, fat depth and marbling scores were collected at the 12$^{th}$ rib.

#### Nutrient Balance

N mass balance was conducted in 12 open-dirt feedlot pens used previously to assess nutritional impacts on nutrient balance in feedlots[2,13]. Steers in each experiment had 29.6 m$^2$ of pen space

**TABLE 1**
**Diet Composition (% of Diet DM) for Experiments 1, 2, and 3 Finishing Diets**

| | Corn bran level | | |
|---|---|---|---|
| Ingredient | 0-bran | 15-bran | 30-bran |
| Corn bran | 0 | 15 | 30 |
| Dry-rolled corn | 75 | 60 | 45 |
| Corn silage | 15 | 15 | 15 |
| Molasses | 5 | 5 | 5 |
| Supplement | 5 | 5 | 5 |
| Composition | | | |
| Crude protein | 11.9 | 11.9 | 12.0 |
| DIP[a] | 6.7 | 7.6 | 8.7 |
| Calcium | 0.65 | 0.65 | 0.65 |
| Phosphorus | 0.23 | 0.21 | 0.18 |

[a]DIP was increased as corn bran increased because microbial efficiency was predicted to increase with higher levels of bran. DIP increased because less feather meal/blood meal was included as bran level increased.

*Note*: Diets for digestibility experiment were similar except 1.5% urea was used to ensure abundant degradable N and 0.25% $Cr_2O_3$ was added as a marker.

and 61 cm of linear bunk space with *ad libitum* access to water. Animals were fed once daily in the morning.

Mass balance procedures were conducted similar to procedures outlined by Bierman et al.[13]. N balance was divided into two separate components: one was conducted from October to May in experiments 1 and 2; experiment 3 was handled separately, with steers fed from June through September. The main reason for combining experiments 1 and 2 was the difficulty in hauling manure and soil sampling pens in February. Time of year can impact N losses due to ambient temperature[2,3]. Mass balance accounting was conducted to assess the impact of dietary treatment on N flow in open-dirt feedlot pens. Briefly, N intake was quantified by accounting for dry matter intake (DMI) and N concentration of dietary ingredients. Feed refusals were quantified, composited, and analyzed to correct N intakes. N excretion was calculated by the difference between N intake and N retained in cattle. N retention in the animal was based on animal performance and weight using retained energy and retained protein equations[12]. These equations are currently the best estimates of N retention; due to the small amount of N retained and the subsequent small impact on N excreted, the errors associated with use of these equations are small.

At the time of slaughter, cattle were removed and the pens scraped. Collected manure was piled on the cement apron and sampled at the time of removal. Wet manure was weighed at time of removal and samples (20 to 25 subsamples corresponding to one subsample per loader bucket) were used to account for nutrients (DM, OM, N) removed in manure. Pens were cleaned in a manner to minimize soil contamination. Because of inherent differences in cleaning from pen to pen and the difficulty in minimizing soil contamination, soil in clean pens was sampled before each experiment and again following cleaning. The soil cores from before and after the nutrient balance experiment were used to correct for either manure left in the pen or soil removed at cleaning. This method allows for accounting of either N addition or loss from pen soil. Soil cores (15-cm depth) were grid sampled at 16 locations within each pen to account for sampling variation. It was assumed that no N movement occurred below 15 cm based on compaction and water movement in feedlot pens[14,15]. Each core accounted for a 14.8-$m^2$-grid area. N in precipitative runoff was also quantified by sampling each runoff event and measuring total volume. Pens are designed to drain into retention ponds, with two pens on the same treatment draining into one pond due to pen design and slope. Runoff volumes were quantified with a flow meter during draining (ISCO 4230 bubbler flow meter, ISCO Inc., Lincoln, NE) and subsamples collected. For each experiment, weighted composite samples were analyzed for total Kjeldahl N[16] and used to calculate total N weight per animal. N in sediment that may have settled out of runoff was accounted for in retention ponds and assumed to be a fraction of runoff. N losses were calculated by difference between N excreted and N in manure, soil core balance, and runoff.

Total N was assayed on feed and feed refusals by combustion method using a N analyzer (LECO FP428, LECO Corporation, St. Joseph, MI). Feed ingredients were composited by month and ground prior to analysis. Feed refusals were composited by pen for each experiment using a weighted average for total DM refused within experiment. Runoff samples were analyzed wet by Kjeldahl N procedure[16]. DM analysis was conducted by drying in forced-air ovens at 60°C for 48 h for all feeds, manure, and soil cores. Manure samples were ground and composited by pen for N analysis. Based on numerous experiments conducted here, ammonium concentration in open-dirt feedlot manure is less than 5% of total N and was not accounted for due to potential loss from oven drying of manure. Soil core samples were ground following drying and composited by pen prior to analysis. Manure and soil core analysis for N was conducted at commercial laboratory using combustion techniques[16] (Ward Laboratories, Kearney, NE). All grinding was conducted using a Wiley mill (1-mm screen).

### Digestibility Trial

Six ruminally and duodenally cannulated steers (BW = 611 kg) were used in a replicated, 3 × 3 Latin square digestibility trial. Surgical and postsurgical care procedures were similar to those outlined by Stock et al.[17]. Diets were similar to diets used in the feedlot except 1.5% urea and 0.25% chromic oxide ($Cr_2O_3$, DM-basis) were provided in the supplement. Steers were fed by automatic feeders with feed provided every 2 h. Steers were housed in 1.5- × 2.4-m individual pens with slotted floors. Pens were cleaned twice daily and room temperatures were controlled and maintained at 25°C. Digestibility was determined using $Cr_2O_3$ as a marker and differences between Cr intake and excretion via feces[18]. Periods were 14 days in duration with feces collected during the last 5 days. Fecal samples were dried in a 60°C forced-air oven (one replicate) or freeze dried (one replicate) for DM determination, ground, and composited by steer within period. Oven-dried fecal samples were analyzed for OM and Cr. N analysis was conducted by combustion method[16] (LECO FP428, LECO Corporation, St. Joseph, MI) on freeze-dried feces. OM analysis was conducted by ashing in a muffle furnace at 600°C for 4 h[16]. Cr analysis was conducted by atomic absorption[19] following ashing and digestion to ensure Cr in solution. Because the digestibility trial was used only to estimate OM excretion in the nutrient balance experiments, N in urine was not quantified.

### Statistical Analysis and Animal Care

Animal care and procedures for the feedlot and metabolism experiments were approved by the University of Nebraska Institute for Animal Care and Use Committee (IACUC approval #98-04-021). Experiments were analyzed as a completely randomized design using GLM procedures of SAS[20]. Animal performance data were tested for experiment by treatment interactions. If no interaction was detected, main treatment effects were evaluated for performance. N mass balance data were analyzed as two components with experiments 1 and 2 analyzed together and experiment 3 separately. Orthogonal contrasts (linear and quadratic) were used to test effects of dietary bran level on performance, digestibility, and N mass balance.

## RESULTS AND DISCUSSION

### Feedlot Performance

No significant interactions between experiment and treatment were detected for performance variables across experiments 1,

2, and 3, which reflects the similar type of cattle used across experiments as well as the same dietary treatments. The only change between experiments was time of year. Therefore, performance data were pooled and are presented in Table 2. Final weight tended to decrease linearly ($p = 0.07$) as bran level increased in the diet, reflecting linear depressions ($p = 0.05$) in average daily gain (ADG). Intakes increased as higher levels of corn bran were fed in place of DRC. Comparing 0-bran to 15-bran, DMI increased 5.1%. DMI increased 6.8% when 0-bran was compared to 30-bran. Because ADG decreased while DMI increased, feed efficiency expressed as ADG:DMI decreased linearly ($p = 0.01$) as bran level increased. Based on feed efficiency, corn bran provided less energy than replaced DRC. Cattle consumed more feed to maintain ADG by offsetting lower energy concentrations in the 15- and 30-bran treatments.

Feed efficiency decreased 7.8% when 0-bran was compared to 15-bran, but decreased only another 2.8% when bran increased from 15 to 30% of diet DM (comparing 15-bran to 30-bran). Surprisingly, these performance data suggest that the second 15% increment was used more efficiently than the first 15% increment of corn bran. Scott et al.[11] evaluated 15 or 30% bran inclusion with DRC-based diets individually fed to yearling steers and observed higher feed efficiency with 15% bran compared to no bran. However, feeding 30% bran slightly (2%) decreased feed efficiency compared to cattle fed the DRC-control diet with no bran[11]. When replacing corn with corn bran, which is less digestible, performance results can mask depressed digestibility because control cattle are experiencing acidosis[21]. Therefore, results from experiments 1, 2, and 3 suggest that cattle fed 0-bran treatments were not experiencing acidosis-related problems and that corn bran negatively impacted performance.

### Nutrient Balance

Because nutrient balance in experiments 1 and 2 had to be conducted together, data for N mass balance are presented as one balance period in Table 3. Because DMI increased as bran inclusion increased while N concentration of diets was similar, N intake increased linearly ($p = 0.01$) as bran increased. N excretion responded similarly to N intake because the N retained by the animal was not impacted by dietary treatment. As the data suggest, most (>90%) of the N fed was excreted based on NRC[12] prediction equations. The steers used in these experiments were large (>380 kg BW), suggesting that fat deposition was large while protein deposition (N retention) was small. The large steers were also fed protein in excess of requirements during the entire experiment. The relatively low retention of N (as % of N fed) agrees with other research[2,13]. Feeding less protein can improve the percentage of N fed that is retained from 10 to 20%[22].

N removed in manure corrected for soil core balance was increased linearly ($p = 0.01$) by increasing dietary corn bran in experiments 1 and 2. Manure N increased 68% when comparing 0-bran to 15-bran and almost doubled (98% increase) when comparing 0-bran to 30-bran. When expressed as a percentage of total N excreted, 25.6, 40.1, and 46.0% of the N was in manure for 0-, 15-, and 30-bran treatments, respectively. N lost via volatilization was also linearly reduced ($p = 0.01$) by increasing dietary bran. Expressed as a percentage of N excreted, 74.1, 59.8, and 53.8% of the N was lost from pens on the 0-, 15-, and 30-bran treatments, respectively. Comparing 0-bran to 15-bran, N losses was reduced by 14.2%. Comparing 0-bran to 30-bran, N losses were reduced by 20.4%. More OM was removed from pens on the higher bran treatments compared to 0-bran. However, despite increased manure N and decreased N losses, neither percent N in manure DM nor C:N ratios of manure were different across dietary treatments. These data suggest that more N was contained in manure for the 15- and 30-bran treatments because more manure was removed. Manure N as a percentage of manure OM was 5.7, 6.3, and 5.5% for 0-, 15-, and 30-bran, respectively. Amount of N lost via precipitative runoff was small (<0.4% of excreted N) relative to N in manure and volatilized N.

In experiment 3, with yearlings fed from June until October, N intakes and N excretion tended to increase linearly ($p = 0.08$) as dietary bran increased (Table 4). As was observed in experiments 1 and 2, the small increase in N intake and excretion with the 15- and 30-bran treatments are related to increased DMI because N concentration in diets were similar. No differences were observed for N in manure, N in runoff, or N volatilized from the pen surface. N losses were not decreased by feeding bran despite linear increases ($p = 0.02$) in the C:N ratio and the OM percentage of manure ($p = 0.08$). Volatile N losses were large and aver-

**TABLE 2**
**Effects of Dietary Corn Bran on Finishing Performance of Yearlings Fed Either 0 (0-Bran), 15 (15-Bran), or 30% (30-Bran) of Diet DM as Corn Bran in Place of DRC**

| | Corn bran level | | | | | | |
|---|---|---|---|---|---|---|---|
| ITEM | 0-bran | 15-bran | 30-bran | SEM | Trial*trt[a] | Linear[b] | Quad[b] |
| Initial wt., kg | 404 | 404 | 404 | 1 | 0.99 | 0.86 | 0.93 |
| Final wt., kg | 612 | 605 | 604 | 3 | 0.47 | 0.07 | 0.48 |
| DMI, kg/d | 11.8 | 12.4 | 12.6 | 0.1 | 0.62 | 0.01 | 0.10 |
| ADG, kg/d | 1.82 | 1.76 | 1.74 | 0.03 | 0.31 | 0.05 | 0.58 |
| ADG/DMI, kg gain/kg feed | .154 | .142 | .138 | 0.002 | 0.10 | 0.01 | 0.09 |

[a]Experiment by bran level interaction.

[b]Linear and Quadratic orthogonal contrasts to corn bran level of 0, 15, and 30%.

*Note*: Data were pooled for experiments 1, 2, and 3 with 96 steers in each experiment and fed for an average of 114 days.

**TABLE 3**
**Effects of Dietary Corn Bran on N Balance in the Feedlot and Manure Characteristics for Steers Fed from October to June (Experiments 1 and 2)**

| | Corn bran level | | | | | |
|---|---|---|---|---|---|---|
| ITEM | 0-bran | 15-bran | 30-bran | SEM | Linear[a] | Quad[a] |
| N intake, kg/steer | 54.4 | 57.9 | 59.5 | 0.5 | 0.01 | 0.20 |
| N excretion, kg/steer | 49.2 | 52.6 | 54.3 | 0.5 | 0.01 | 0.24 |
| N manure, kg/steer[b] | 12.6 | 21.1 | 25.0 | 1.6 | 0.01 | 0.28 |
| N runoff, kg/steer | 0.20 | 0.09 | 0.06 | 0.01 | 0.01 | 0.01 |
| N volatilization, kg/steer[c] | 36.7 | 31.5 | 29.2 | 1.8 | 0.03 | 0.52 |
| % volatilization[d] | 74.1 | 59.8 | 53.8 | 3.2 | 0.01 | 0.33 |
| % N manure[e] | 1.80 | 1.69 | 1.76 | 0.11 | 0.83 | 0.55 |
| C:N manure[f] | 13.5 | 14.3 | 14.4 | 0.7 | 0.41 | 0.67 |
| OM manure, kg/steer | 222 | 335 | 455 | 17 | 0.01 | 0.86 |

[a]Linear and Quadratic orthogonal contrasts to corn bran level of 0, 15, and 30%.
[b]Manure N is corrected for change in pen soil N concentration and N amount from before and after experiments.
[c]Volatilization calculated as N excretion-N manure-N from soil balance-N in runoff
[d]Percent volatilization expressed as a percent of N excretion
[e]Nitrogen concentration of manure removed at cleaning expressed as % of manure DM.
[f]Carbon to nitrogen ratio of manure removed at cleaning.

*Note*: Data were combined for both experiments and handled as one nutrient balance period. Nutrient balance data for N are expressed as total kg per steer for both experiments (233 days).

**TABLE 4**
**Effects of Dietary Corn Bran on N Balance in the Feedlot and Manure Characteristics for Steers Fed from June to October**

| | Corn bran level | | | | | |
|---|---|---|---|---|---|---|
| ITEM | 0-bran | 15-bran | 30-bran | SEM | Linear[a] | Quad[a] |
| N intake, kg/steer | 24.7 | 25.7 | 26.0 | 0.4 | 0.08 | 0.53 |
| N excretion, kg/steer | 22.5 | 23.5 | 23.7 | 0.4 | 0.07 | 0.50 |
| N manure, kg/steer[b] | 6.6 | 7.6 | 7.2 | 1.3 | 0.76 | 0.70 |
| N runoff, kg/steer | 1.04 | 1.02 | 1.09 | 0.14 | 0.82 | 0.79 |
| N volatilization, kg/steer[c] | 14.9 | 16.3 | 15.5 | 1.5 | 0.79 | 0.57 |
| % volatilization[d] | 66.3 | 69.2 | 65.0 | 5.9 | 0.88 | 0.63 |
| % N manure[e] | 1.33 | 1.13 | 1.34 | 0.13 | 0.94 | 0.22 |
| C:N manure[f] | 12.6 | 13.5 | 14.0 | 0.4 | 0.02 | 0.61 |
| OM manure, kg/steer | 146 | 168 | 182 | 13 | 0.08 | 0.81 |

[a]Linear and Quadratic orthogonal contrasts to corn bran level of 0, 15, and 30%.
[b]Manure N is corrected for change in pen soil N concentration and N amount from before and after experiments.
[c]Volatilization calculated as N excretion-N manure-N from soil balance-N in runoff
[d]Percent volatilization expressed as a percent of N excretion
[e]Nitrogen concentration of manure removed at cleaning expressed as % of manure DM.
[f]Carbon to nitrogen ratio of manure removed at cleaning.

*Note*: Nutrient balance data for N are expressed as total kg per steer for the entire experiment.

aged 66.8% of total N excreted. Approximately 30.7% of excreted N was removed in manure at cleaning across dietary treatments. Runoff N was greater in experiment 3 than in experiments 1 and 2, averaging 4.5% of total N excreted. The runoff amounts observed in experiment 3 agree with previously published averages of 3 to 6% of nutrient excreted[2,13,23,24]; however, little runoff occurred during experiments 1 and 2 because of low precipitation.

Increasing the C:N ratio by increasing dietary bran had variable impacts on N losses in these experiments. During the colder winter–spring months (experiments 1 and 2), N losses were markedly decreased by adding corn bran to feedlot diets. However, small differences in N losses were observed between treatments in experiment 3. Dewes[3] evaluated N losses from cattle manure in chambers by studying temperature and C additions separately. Increasing ambient temperature resulted in rapid (within

4 days) losses at high temperatures (40°C) whereas losses at temperatures of 20°C were still large but much slower[3]. In experiment 3, increasing the C:N ratio of manure by dietary manipulation in the summer may not influence N losses because of the rapid losses with higher temperature. Based on average high and low temperatures for these experiments, the average temperature for experiments 1 and 2 was 6.0°C whereas average temperature for experiment 3 was 23.1°C.

Another observation from these experiments is that the amount of N lost from pens in the 0-bran treatment was higher (74.1% of N excreted) for experiments 1 and 2 compared to experiment 3 (66.3% of N excreted). Despite colder average ambient temperatures during experiments 1 and 2, just as much N was lost from pens on the same diet as was lost in experiment 3. This observation suggests an interaction between diet type (C:N ratio of manure) and temperature. It appears that if adequate C is present when temperatures rise in May, N losses may be minimized. However, if inadequate C is present (0-bran), then N losses will be just as large as continuous warm temperatures.

Other research has given variable results when C is added to manure. Andersson[4] added rapidly degraded glucose and slowly degraded straw and peat to liquid hog manure to determine the impact on N losses. Glucose decreased N losses during the initial 8 days. However, adding straw and peat decreased N losses more (by 15 times) and longer (7 weeks) compared to untreated and glucose-amended hog manure. Subair et al.[5] added either 2.5 or 5.0% paper products to hog manure and monitored volatilization. In their study, adding paper decreased N losses from 53 to 28%. In both the Andersson and the Subair et al. studies[4,5] manure was stored under aerobic conditions. When C added to manure was evaluated under anaerobic conditions, variable results were observed with some decreasing N losses[25,26] and some having no effect[27].

None of these studies were conducted either to evaluate dietary modifications to increase C:N ratio or with open-dirt feedlot pens. Bierman et al.[13] evaluated diets containing no roughage, 7.5% roughage, and 7.5% roughage with 40% wet corn gluten feed (WCGF) fed to steers in open-dirt feedlot pens. WCGF fed at 40% would be similar to diets containing approximately 27% corn bran based on the source of WCGF in their study. However, dietary N concentration was not equivalent across treatments. Despite different N intakes, N removed in manure was improved by feeding roughage and roughage with WCGF. In a similar experiment with open-dirt feedlot pens, corn silage increased in the diet from 15 to 45% had no impact on N losses[2]. Presumably, corn silage fiber is less available to microbes on the pen surface because of ensiling and feeding as compared to the corn bran used in these experiments. Corn bran may pass through the rumen more quickly due to smaller particle size than corn silage and may stimulate more C excretion in the feces as compared to corn silage. Based on previous literature and these results, C additions to manure either through the diet or by direct addition may have variable results on N losses due to how rapidly degradation occurs.

Rainfall was different across these two time periods as well (experiments 1 and 2 vs. experiment 3). During experiment 3, there was 27.4 cm of precipitation during the 110 days. In experiments 1 and 2, precipitation totaled 19.0 cm over 233 days. The increased moisture from 8.4 cm of precipitation in less than half as many days for experiment 3 as compared to experiments 1 and 2 may have obscured differences in N loss between treatments in experiment 3. Numerous researchers have concluded that N volatilization is positively correlated with moisture content and is most rapid during drying cycles[28,29].

### Digestibility Trial

Cannulated steers used in the digestibility trial consumed 9.8 kg of DM per day, but DMI was not affected by dietary treatment (Table 5). In the feedlot experiments, DMI increased linearly and tended to increase quadratically as dietary bran increased. Based on marker concentrations in feces, DM digestibility decreased linearly ($p = 0.07$) as corn bran increased from 0 to 30% of diet DM. Similarly, OM digestibility decreased linearly ($p = 0.07$) from 77.3 to 73.1% of OM intake. Scott et al.[10] evaluated DRC-based diets with or without 15% corn bran in a total fecal collection digestion trial and observed a decrease in DM digestibility from 84.5 to 80.3% when bran was added.

**TABLE 5**
**DM, OM, and N Digestibility Results from Replicated Latin Square Digestibility Trial Using Ruminally and Duodenally Cannulated Steers**

| | Corn bran level | | | | | |
|---|---|---|---|---|---|---|
| ITEM | 0-bran | 15-bran | 30-bran | SEM | Linear[a] | Quadratic[a] |
| DM Intake, kg/day | 9.7 | 10.1 | 9.7 | 0.2 | 0.81 | 0.23 |
| DM digestibility, % | 75.8 | 74.3 | 71.7 | 1.4 | 0.07 | 0.75 |
| OM Intake, kg/day | 9.2 | 9.6 | 9.2 | 0.2 | 0.87 | 0.23 |
| OM digestibility, % | 77.3 | 75.9 | 73.1 | 1.5 | 0.07 | 0.70 |
| N intake, grams/day | 194 | 209 | 208 | 5 | 0.08 | 0.24 |
| N excreted, grams/day | | | | | | |
| In feces | 61 | 66 | 70 | 3 | 0.05 | 0.80 |
| N digestibility, % | 68.7 | 68.3 | 66.4 | 1.4 | 0.11 | 0.51 |

[a]Linear and Quadratic orthogonal contrasts to corn bran level of 0, 15, and 30%.

More N was excreted in the feces (70 vs. 61 g/day) for steers fed 30-bran compared to 0-bran, suggesting that route of excretion for N may have been affected by dietary treatment. Increasing fiber inclusion in corn-based diets may change route of N excretion from urine to feces by stimulating hindgut fermentation[13,30]. Presumably, corn bran would increase hindgut fermentation compared to 0-bran diets comprised of corn and 15% corn silage. Corn bran contains between 70 and 86% neutral detergent fiber[9,31]. Bran used in these experiments averaged 81.3 ± 1.3% neutral detergent fiber. Bierman et al.[13] changed route of excretion from urine to feces when a 40% WCGF diet was compared to a 7.5% roughage diet similar to the 0-bran diet fed in this experiment. Because WCGF is comprised of corn bran and corn steep from the wet milling industry, corn bran alone may have similar effects on route of excretion. Bran is probably the sole stimulant of hindgut fermentation in WCGF-based diets because steep is more digestible than corn[31].

### CONCLUSIONS

Increasing the C:N ratio of feedlot manure by dietary manipulation may have value in decreasing N losses, but it is dependent on time of year. However, nutritional methods that increase the C:N ratio of manure will lead to decreases in feed efficiency that may limit their adoption and usefulness for producers. Corn bran may offer value in minimizing N losses; however, decreasing digestible OM will depress performance. N losses during the summer months are a concern and are not easily controlled by changing the C:N ratio of manure.

| | | |
|---|---|---|
| Received: | October | 9, 2001 |
| Revised: | November | 6, 2001 |
| Accepted: | November | 7, 2001 |
| Published: | November | 21, 2001 |
